# Supplementary material for: Tumor Suppressive Circular RNA-102450: Development of a Novel Diagnostic Procedure for Lymph Node Metastasis from Oral Cancer
Source: Cancers (Basel). 2021 Nov 15;13(22):5708. doi: 10.3390/cancers13225708 (PMC8616294; doi:10.3390/cancers13225708)
Supplement: Supplementary file 1 [file cancers-13-05708-s001.zip › cancers-1432590-supplementary.pdf]

cancers

MDPI

Supplementary Materials: Tumor Suppressive Circular RNA-102450: Development of a Novel Diagnostic Procedure for Lymph Node Metastasis from Oral Cancer

Toshiaki Ando, Atsushi Kasamatsu, Kohei Kawasaki, Kazuya Hiroshima, Reo Fukushima, Manabu Iyoda, Dai Nakashima, Yosuke Endo-Sakamoto and Katsuhiro Uzawa

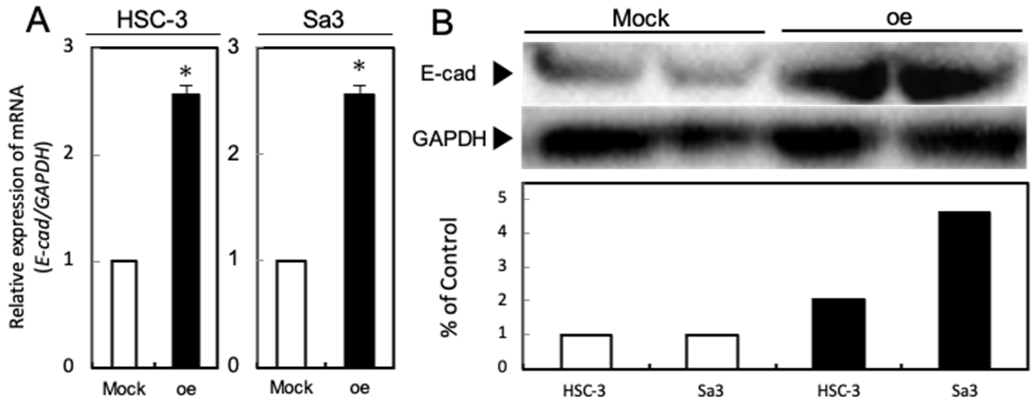

Figure S1. E-cadherin (E-cad) mRNA (**A**) and protein (**B**) expressions in oe-circRNA-102450 and mock cells (derived HSC-3 and Sa3) (n = 3)(* $p < 0.05$ is significant.).

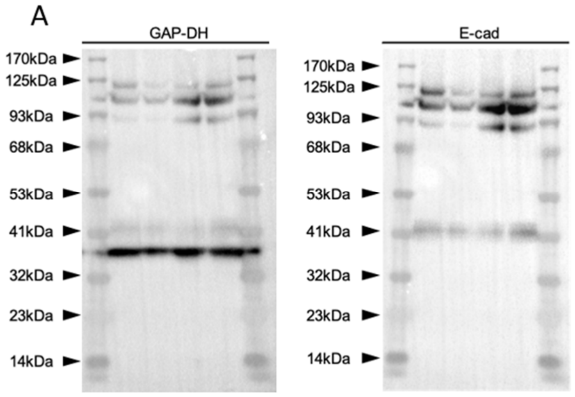

B

	Densitometry Readings		Intensity Ratio
	E-cad	GAPDH	E-cad/GAPDH
HSC-3 Mock	1829677	2281044	0.802122625
Sa3 Mock	886795	1946456	0.455594681
HSC-3 oe	3221871	1976049	1.630461087
Sa3 oe	4048598	1924539	2.103671581

Figure S2. Full length blots of Figure S1 (**A**) and densitometry readings/intensity ratio of each band (**B**).
